# Molecular Phylogeography and Population Genetic Structure of an Endangered Species *Pachyhynobius shangchengensis* (hynobiid Salamander) in a Fragmented Habitat of Southeastern China

**DOI:** 10.1371/journal.pone.0078064

**Published:** 2013-10-18

**Authors:** Yanyu Zhao, Yanhua Zhang, Xiaochen Li

**Affiliations:** 1 College of Life Sciences, Shaanxi Normal University, Xi'an, China; 2 College of Life Sciences, Jiangsu Normal University, Xuzhou, China; Auburn University, United States of America

## Abstract

The salamander *Pachyhynobius shangchengensis* (Hynobiidae) is a vulnerable species restricted to a patchy distribution associated with small mountain streams surrounded by forested slopes in the Mount Dabieshan region in southeastern China. However, molecular phylogeography and population genetic structure of *P. shangchengensis* remain poorly investigated. In this study, we explored the genetic structure and phylogeography of *P. shangchengensis* based on partial sequences of the mitochondrial DNA (mtDNA) cytochrome b and cytochrome c oxidase subunit I genes. Fifty-one haplotypes and four clades were found among 93 samples. Phylogenetic analyses revealed four deeply divergent and reciprocally monophyletic mtDNA lineages that approximately correspond to four geographic regions separated by complicated topography and long distances. The distinct geographic distributions of all lineages and the estimated divergence time suggest spatial and temporal separation coinciding with climatic changes during the Pleistocene. Analysis of molecular variance indicated that most of the observed genetic variation occurred among the four groups, implying long-term interruption of gene flow, and the possible separation of *P. shangchengensis* into four management units for conservation.

## Introduction

Molecular ecology primarily aims to understand the influence of abiotic factors, such as altitude, topography, and glacial history, on the spatial distribution of genetic variations [1]. Recently, researchers have studied the effect of landscape variables, such as topography and altitude, on the geographical distribution of genetic variation in the emerging field of landscape genetics [2]. Landscape characteristics can affect the proportion of suitable habitats, migration patterns, and the genetic divergence of populations [2,3]. For terrestrial species, especially for amphibian, species diversification drived by landscape variables can occur by the formation of sky islands in which hot, dry, deep valleys serve as barriers to gene flow [4], as well as the height of mountains forming a barrier to dispersal for amphibian that live in the valleys [5]. Mountain ridges have also been shown to be an important barrier to amphibian dispersal and gene flow [2,6]. Therefore, a complex, microhabitat-rich topography, could effect genetic diversity and phylogeographic structure of animal habitats in these areas [7].

Using phylogeographical tools to analyze the effects of landscape characteristics on species distributions over large spatial scales has provided remarkable insight into the spatial patterns of genetic diversity [8]. Using highly variable genetic markers and a dense sampling regime across a small, topographically diverse region enables investigation of the localized effects of geography on genetic diversity and connectivity across the landscape [1]. 

Besides landscape characteristic, climatic changes have also caused montane species to expand, change, or be in contact with each other along latitudinal or elevational gradients associated with Pleistocene glacial cycles [9–11]. “East Asia is characterized by a mosaic of mountains and likely experienced a relatively mid-Pleistocene climate” [12]. The Dabieshan Mountains are connected to the eastern end of the Tsinling Mountains by the Tongbaishan Mountains, and are located in the eastern part of China. Though most mountains in this area were not glaciated during the Pleistocene [13,14], this region experienced climatic fluctuations which probably impacted species distributions, demography and diversification [15]. Phylogeography and population genetic structure of *P. shangchengensis* which lives in here should be affected by climatic changes. 

Shangcheng Stout Salamander *P. shangchengensis* (Hynobiidae) is an endemic species in china, its distributions restrict in the Mount Dabieshan regions, which was described by Fei et al. [16] from Mount Huangbaishan, Shangcheng County, Henan Province (holotype) and Mount Jingangtai, Shangcheng County (paratypes). It can be found in patchy habitat on the Dabieshan Mountains, southeastern China [17,18]. *Pachyhynobius shangchengensis* has low vagility, its habitat is separated by valleys and low lands, and its distributions are getting smaller and smaller [17]. Chen et al. [19] described *Hynobius yunanicus* based on specimens from Huangbaishan, Shangcheng County. *Hynobius yunanicus* differs from *P. shangchengensis* mainly in having little white spots on deep brown dorsal side, in lacking premaxillary fontanelle on the skull, and in lacking connection between maxillary and pterygoid. However, evidences from karyotypic and phylogenetic analysis rejected the validity of *H. yunanicus* [20]. Therefore, *Hynobius yunanicus* is a synonym of *P. shangchengensis*, and our sampling sites should include Huangbaishan.

**Figure 1:**
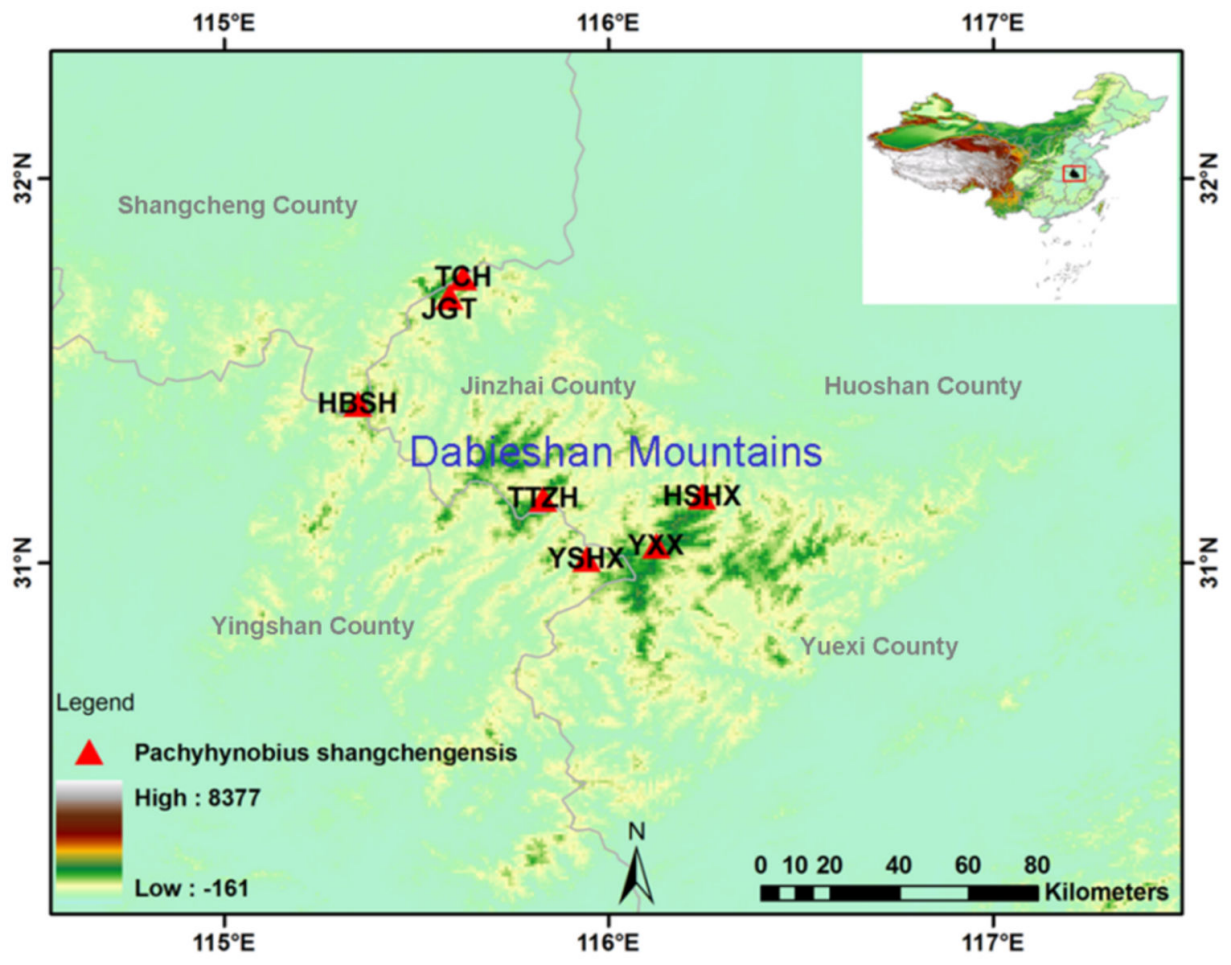
Distributions of *Pachyhynobius shangchengensis*. Sampling sites for the present study are marked by red triangles and coded names (Table 1).

 From 2011–2012, the authors of this paper investigated the geographical distribution of *P. shangchengensis*, the sampling sites include Jingangtai (JGT), Tiechong (TCH), Tiantangzhai (TTZH), Huangbaishan (HBSH), Yingshanxian(YSHX), Yuexixian (YXX) and Huoshanxian (HSHX). The above sampling sites are isolated from each other by more than 20 kilometers (Figure 1). 

In this study, we test whether the topography of the mountains affected the population genetic structure of the *P. shangchengensis* according to partial sequences of the mitochondrial DNA cytochrome b (mtDNA cyt *b*) and cytochrome c oxidase subunit I (mtDNA *COI*) genes. We also determined whether climatic oscillations during glacial periods in the Quaternary affected the distribution of *P. shangchengensis*. 

## Materials and Methods

### Sampling, DNA extraction, polymerase chain reaction, and sequencing

This study was approved by the Institutional Animal Care and Use Committee (IACUC) of Shaanxi Normal University and Chinese Academy of Sciences. A total of 93 *P. shangchengensis* individuals were collected from seven locations on the Dabieshan Mountains, Southeast China (Table 1; Figure 1). Samples were obtained upon capture by toe clipping from live specimens, which were subsequently released.

**Table 1:** Sampling locations and haplotypes with frequencies and genetic diversities.

Sampling sites	GPS coordinates	Elev.(m)	SS	Haplotypes and their frequencies	*Pi±SD*	*Hd±SD*
JGT	115.58540E 31.69173N	780	12	Hap28(7), Hap29(1), Hap30(1), Hap31(3)	0.00065±0.00051	0.636±0.128
TCH	115.62168E 31.74129N	829	14	Hap1(1), Hap2(2), Hap3(1), Hap4(1), Hap5(1),Hap6(1), Hap7(2), Hap8(1), Hap9(1)	0.00130±0.00193	0.912±0.059
HBSH	115.34738E 31.41311N	716	16	Hap10(1), Hap11(2),Hap12(2), Hap13(4), Hap14(1), Hap15(2),Hap16(1), Hap17(1), Hap18(1), Hap19(1)	0.00142±0.00201	0.925±0.047
TTZH	115.82928E 31.16722N	897	16	Hap32(1), Hap33(7), Hap34(3), Hap35(1), Hap36(1),Hap37(1), Hap38(2)	0.00353±0.00309	0.792±0.089
YSHX	115.94302E 31.01085N	1145	11	Hap39(1), Hap40(3), Hap41(1), Hap42(1), Hap43(1),Hap44(1), Hap45(1), Hap46(1), Hap47(1)	0.00228±0.00315	0.945±0.066
YXX	116.12362E 31.04441N	877	10	Hap40(2), Hap48(4), Hap49(2), Hap50(1), Hap51(1)	0.00213±0.00217	0.822±0.097
HSHX	116.24244E 31.17237N	825	14	Hap20(3), Hap21(1), Hap22(3), Hap24(3), Hap25(1),Hap26(1), Hap27(1)	0.00248±0.00306	0.901±0.052
Total			93		0.0324±0.0203	0.978±0.005

SS, sampling size; Pi, nucleotide diversity; Hd, haplotype diversity; S.D., standard deviation.

Samples were also permitted by following authorities: management committee of Jingangtai Nature Protection Area; management committee of Shangcheng Stout Salamander Nature Protection Area (Henan Shangcheng); management committee of Tiantangzhai National Forest Park; management committee of Yaoluoping Nature Protection Area.

Tissue samples were preserved in 95% ethanol and stored at -20 °C. Total genomic DNA was extracted through a standard phenol: chloroform method [21]. A continuous fragment (942 bp) of the mitochondrial cytochrome b gene was amplified by polymerase chain reaction (PCR) (MyCycler Thermal Cycler), with outer primers L_1_, H_1_ and inner primers (nested primer) L_2_, H_2_ (Table 2). A continuous fragment (1011bp) of the mitochondrial cytochrome c oxidase subunit I (*COI*) was amplified by PCR (MyCycler Thermal Cycler), with forward primer FZ and reverse primer RZ (Table 2). The PCR products were checked in a 1% agarose gel and purified through a TIANquick Midi Purification Kit (Tiangen, Beijing, China) according to protocol recommendations. Sequencing reactions were performed with the PCR primers through an ABI Prism BigDye Terminator Cycle Sequencing-Ready Reaction Kit on an ABI 3730XL sequencer. All sequences were deposited in the GenBank database under accession numbers KC162002-KC162083 (*COI*) and KC162084-KC162162 (*cyt b*).

**Table 2:** PCR conditions and primers to amplify two mitochondrial DNA fragments.

Primer name	5′=> 3′ sequence	Origin	Ann. - T. (℃)	DNAfragment length/gene (abbreviation in text)	Best fit model of sequence evolution
L_1_	CCCAATTCGAAAAACTCACC	This paper	52	Ca.1011bp, mtDNA, Cytochrome b (Cyt b)	GTR+I+G
H_1_	TATAGGGTTGATGCGGCTTG	This paper	52		
L_2_	TTCGTAGATCTCCCAACTCC	This paper	52		
H_2_	CCAATTCAAGTTAAGATTAA	This paper	52		
FZ	ATTTAGTATTTGGTGCCTGAGCTG	This paper	55	Ca. 942 bp, mtDNA Cytochrome Oxidase I gene (COI)	GTR+I+G
RZ	ATCAATGGACAAACC CACCTAT	This paper	55		

All mtDNA PCRs were performed with 94 °C, 1 min; 38×(94 °C 30 s, annealing temperature 1 min; 72 °C lmin) ; 72 °C 5 min.

### Nucleotide polymorphism

The sequences were aligned with Clustal X1.83 [22]. The aligned sequences were edited using the program BioEdit 7.0.9.0 [23]. Haplotype inference was conducted through Collapse 1.2 (http://darwin.uvigo.es). To examine whether the two analyzed regions (cyt *b* and *COI*) can be combined into a larger data matrix [24], we performed a partition-homogeneity test using 1000 replicates as implemented in PAUP 4.10b [25]. The combined data were further analyzed because the result of the partition-homogeneity test was not significant.

The number of variable and parsimony-informative sites was determined using the program DnaSP 5.10.01 [26], and haplotype diversity (*H*_d_) and nucleotide diversity (P_i_) were determined through Arlequin 3.5.1.2 [27].

### Phylogenetic structure

 The phylogenetic relationship among haplotypes was estimated through maximum likelihood (ML) analyses in PAUP*4.0b10 [25], as well as Bayesian analyses in MrBayes 3.0 [28] with 3,000,000 generations. For the Bayesian analyses, MrModelTest 2 [29] was used to find the best-fit substitution model, and GTR+I+G model was performed. For the maximum likelihood analyses, MODELTEST [30] was used to find the best-fit substitution model, and GTR+I+G model was performed. The confidence level of ML trees was accessed by 1000 bootstrap replications. *Hynobius chinensis* and *Hynobius guabangshanensis* were used as the outgroup. The *COI* and cyt *b* sequences of *H. chinensis* were downloaded from GenBank with accession number HM036353, and the *COI* and cyt *b* sequences of *H. guabangshanensis* were downloaded from GenBank with accession number EF616473 (*cyt b*) and FJ913877 (*COI*). 

 We also used NETWORK 4.5.0.2 [31] to draw a median-joining network to analyze the relationships among detected haplotypes.

### Analyses of geographic structuring

 The population and phylogroup comparisons using pairwise difference and the partitions of genetic diversity within and among populations were analyzed through analysis of molecular variance (AMOVA) [32] using Arlequin 3.5.1.2 [27] with 10,000 permutations. 

 The spatial genetic structure of haplotypes was analyzed through SAMOVA 1.0 [33] (http://web.unife.it/progetti/genetica/Isabelle/samova.html) with 1000 permutations. The number of initial conditions was set to 100 as recommended by Dupanloup et al. [33]. The number K of groups of populations was set to from 2 to 6 respectively. The K with the highest FCT represents the best number of groups and the best population conFigureuration. This program implements an approach to define groups of populations that are geographically homogeneous and maximally differentiated from each other. The method is based on a simulated annealing procedure that aims to maximize the proportion of total genetic variance caused by differences between groups of populations (FCT).

### Divergence time estimate

The approximate divergence times were estimated for the lineages for *P. shangchengensis* in BEAST 1.6.1 [34]. Except those for outgroups, all haplotype sequences were used in the analysis. A Bayesian Markov chain Monte Carlo approach with an uncorrelated log-normal relax molecular clock was used in BEAST 1.6.1 [34]. Two independent runs were performed, each of which was composed of 120 million generations, with sampling every 1000 generations. A burn-in was set to 10% of the samples. To check for stationarity, the results were displayed in TRACER version 1.5 [35]. LogCombiner 1.4.7 [36] was used to combine both runs. TreeAnnotator 1.4.7 [36] was used to annotate tree information, and FigureTree 1.1.1 [37] to visualize tree information. 

A molecular evolutionary rate of the mitochondrial genome for hynobiids (0.64% per Myr per lineage) was proposed by Weisrock et al. [38]. This evolutionary rate was frequently used for hynobiids mitochondrial DNA data [39–42]. Thus, we used 0.64% per Myr per lineage to estimate the divergence between any major clades.

### Pattern of isolation by distance

 Mantel tests [43] were conducted in Arlequin 3.5.1.2 [27] to assess the significance of isolation by distance between populations with 5000 random permutations on matrices of pairwise population *F*_ST_ and the geographical distances. Pairwise *F*_ST_ values between populations were estimated through Arlequin 3.5.1.2 [27], whereas straight line geographical distances between populations were calculated online at http://www.gpsvisualizer.com/calculators.

### Demographic history analysis

We applied Neutrality tests through the program Arlequin 3.5.1.2 [27] as an assessment of possible population expansion. Under the assumption of neutrality, a population expansion produces a large negative value of Fu’s *F*_S_ test [44] and Tajima’s *D* [45]. Tajima’s *D* and Fu’s *F*_S_ are sensitive to bottleneck effects or population expansion, causing these values to be more significantly negative [46–49]. Fu’s *F*_S_ is particularly sensitive to recent population growth [44]. 

 Population expansion events were determined through mismatch analysis [50] using Arlequin 3.5.1.2 [27], with the number of bootstrap replicates set to 5000 to explore the demographic history of the studied populations. The parameters of demographic expansion were also estimated. Recent growth is expected to generate a unimodal distribution of pairwise differences between sequences [50]. The validity of the expansion model was tested by using the sum of squared deviations (SSD) and Harpending’s raggedness index (*R*) between observed and expected mismatches. The formula T=τ/(2ut) was used to estimate the time of the population expansions [50] based on the generation time (3.5 years) [51], *t* is the date of growth or decline (mutational time), τ is the mode of mismatch distribution (evolutionary time), and *u* is the mutation rate per sequence and per generation [52]. 

 The Bayesian skyline plot (BSP) was used to estimate the demographic history of *P. shangchengensis* using the program BEAST 1.6.1 [34]. A piecewise-constant skyline model was selected, and a relaxed uncorrelated log-normal molecular clock was used with the mutation rate of 0.64%/MY for *P. shangchengensis* as suggested by Weisrock et al. [38]. Tracer 1.5 was used to reconstruct the demographic history through time.

## Results

### Genetic variation

The total number of sites (excluding sites with gaps/missing data) was 1953, of which 1011 bp were sequenced for the *COI* gene and 942 bp for the cyt *b* gene. A total of 195 polymorphic sites were found, of which 182 were parsimony-informative and 13 were singleton-variable. These polymorphic sites identiﬁed 51 haplotypes within 93 individuals from seven localities (Table 1; Figure 1). Each sampled population and the total population have high *H*_d_, accompanied by very low P_i_ (Table 1). 

### Phylogenetic diversity

**Figure 2:**
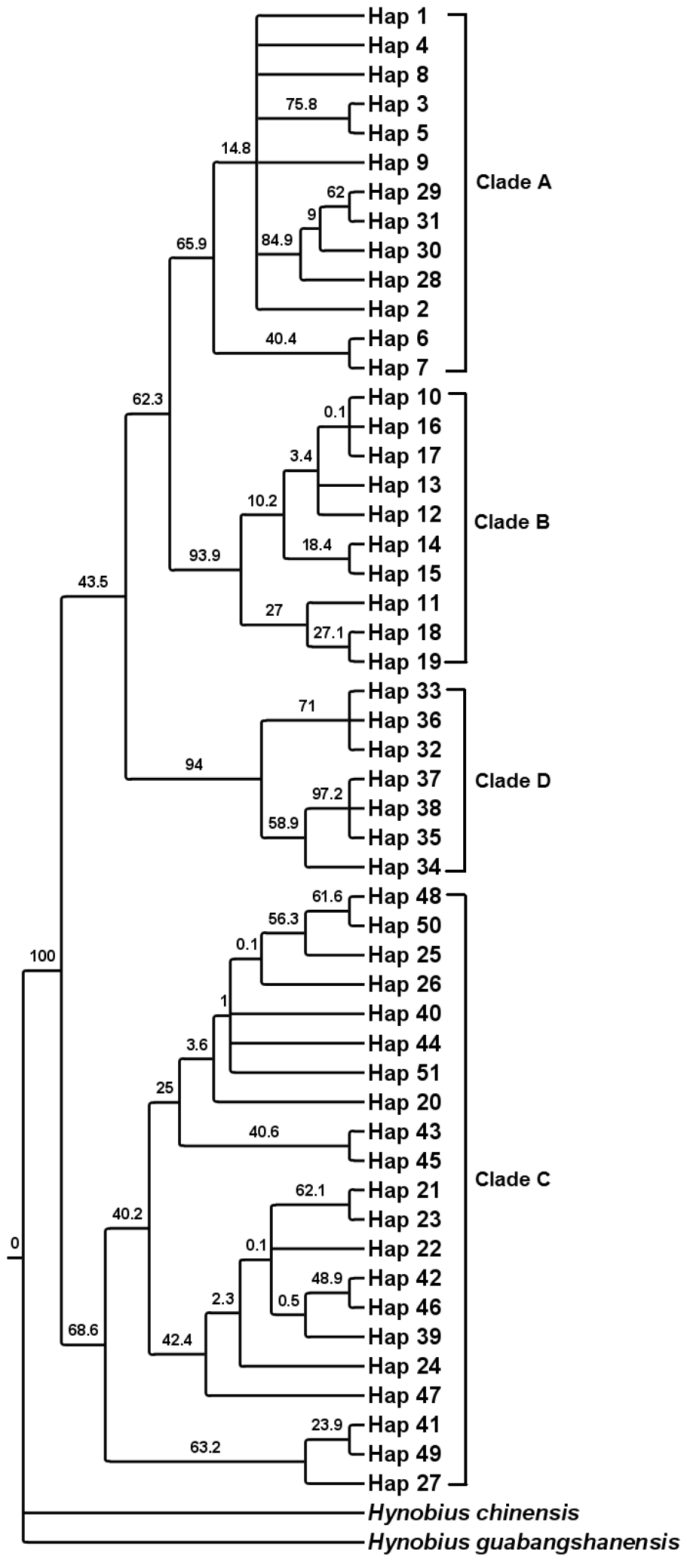
Maximum likelihood (ML) tree of the observed haplotypes of *Pachyhynobius shangchengensis*, with *Hynobius chinensis* and *Hynobius guabangshanensis* as outgroup. Numbers above the branches represent the bootstrap values.

**Figure 3:**
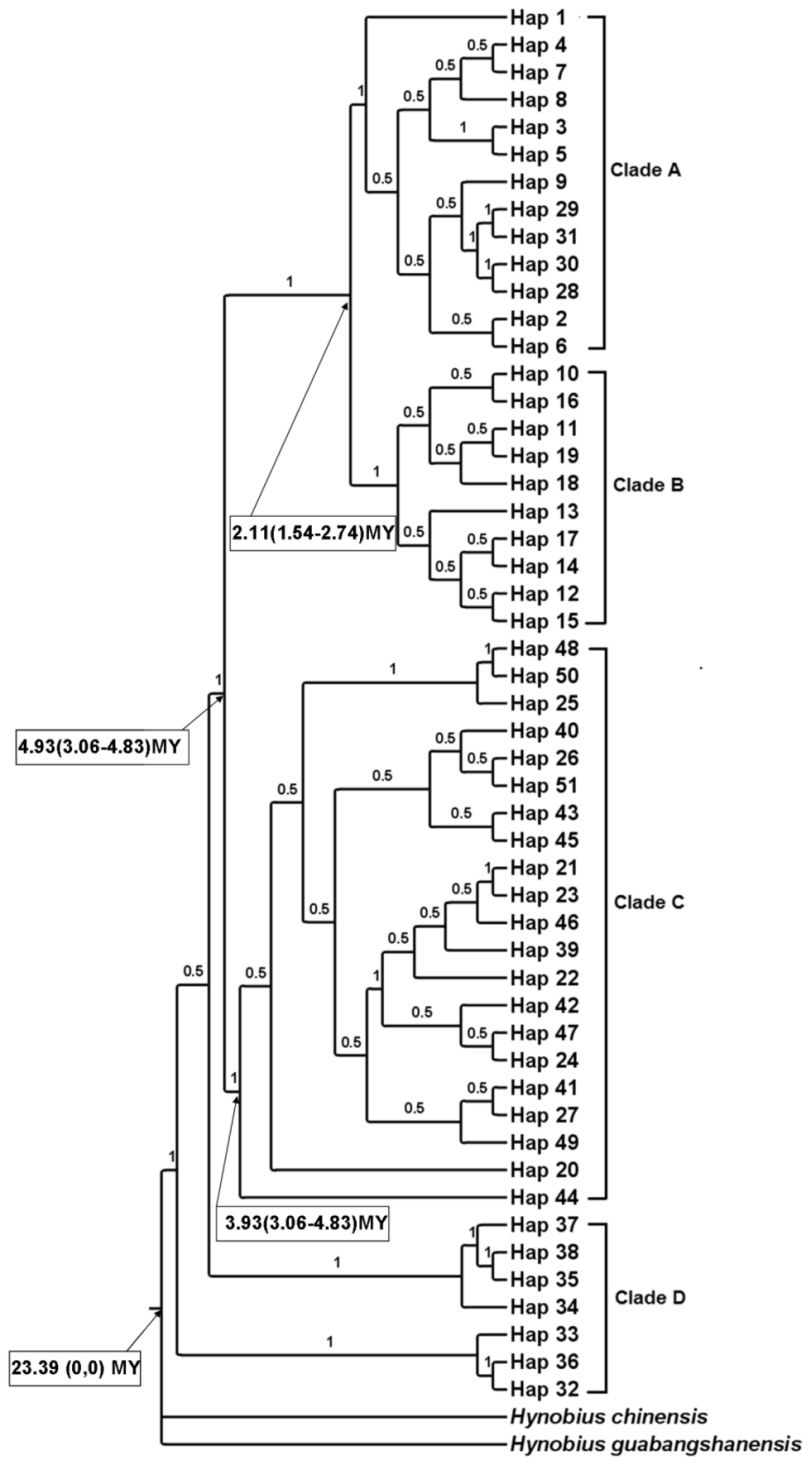
Phylogram of *Pachyhynobius shangchengensis* mtDNA haplotypes obtained with Bayesian in MrBayes, rooted with two sequences from *Hynobius chinensis* and *Hynobius guabangshanensis*. MtDNA clades and estimated age (in MY) obtained with BEAST were indicated. Numbers above nodes, Bayesian posterior probability; numbers below nodes, estimated age and 95% confidence intervals (shown in parenthesis).

**Figure 4:**
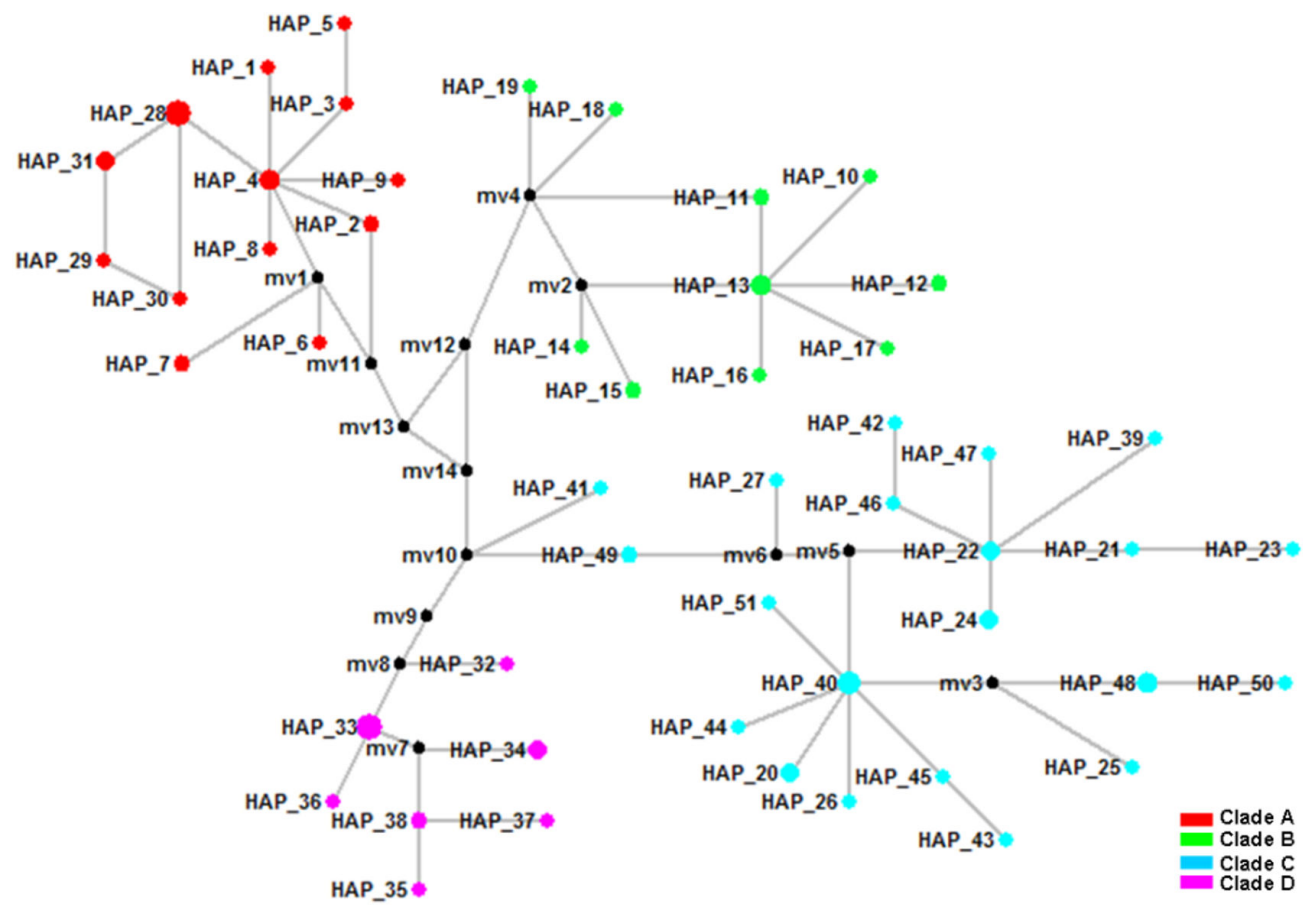
Median-joining network of mtDNA haplotypes of *Pachyhynobius shangchengensis* on the Mount Dabieshan in China. Each haplotype is represented by a circle, with the area of the circle proportional to its frequency. Samples from Clade A to D were indicated by different colours. Median vector (mv1-mv14) is indicated by black.

In both Bayesian and ML phylogenetic analyses, the 51 haplotypes of *P. shangchengensis* observed in the combined dataset formed four distinct clades (Table 1; Figures 2, 3). Clade A, B, C, D, which includes all individuals, collected from JGT–TCH, HBSH, TTZH, and YSHX–YXX–HSHX, respectively (Figures 2, 3). In the haplotype median-joining network, the 51 haplotypes of *P. shangchengensis* observed in the combined dataset formed four distinct clades too (Figure 4).

### Population and geographic structure

Analysis of molecular variance indicated that most of the observed genetic variation occurs among the four groups (JGT–TCH, HBSH, TTZH and YSHX–YXX–HSHX) (93.92%), whereas differentiation among seven endemic populations (JGT, TCH, HBSH, TTZH, YSHX, YXX, HSHX) within groups only contributed 1.48% to the total population, and differentiation within seven endemic populations contributed 4.6% to the total population (Table 3).

**Table 3:** Results of analysis of molecular variance (AMOVA).

Source of variation	*d.f.*	Sum of squares	Variance components	Percentage of variation	Fixation Index (*p*-value)
Among groups	3	2716.443	40.01627 Va	93.92	*F*_ST_ = 0.95402 *p* (rand. value ≤ obs. value) =0.0±0.0
Among Populations within groups	3	28.577	0.63040 Vb	1.48	
Within populations	86	168.464	1.95888 Vc	4.6	
Total	92	2913.484	42.60555		

d.f., degrees of freedom.

For the spatial AMOVA, with *K* increased from 2 to 6, the *F*_CT_ value was highest (*F*_CT_ = 0.9421) when *K* = 4. Thus, the SAMOVA tests revealed the number of significant phylogeographic groups (*K* = 4) .

### Pattern of isolation by distance

Mantel test results showed significant correlation between the pairwise calculated genetic distance and pairwise calculated straight line geographical distance of the populations (correlation coefficient = 0.6406, *p* < 0.001), indicating the presence of isolation-by-distance. This finding suggests that the distribution of genetic variation is due to geographical separation. The Mantel test results provided evidence for large-scale geographical population structure in this species.

### Population Comparisons

Population comparisons showed more or less significant genetic differentiation (FST) between most local populations (Table 4). Thus, a long-term interruption of gene flow among all clades was also evidenced by the relatively high FST values.

**Table 4:** FST values between populations.

Population	TCH	JGT	HBSH	TTZH	YXX	HSHX	YSHX
TCH	0						
JGT	0.53762**	0					
HBSH	0.94272**	0.95588**	0				
TTZH	0.95065**	0.95510**	0.95632**	0			
YXX	0.96245**	0.97049**	0.96577**	0.92695**	0		
HSHX	0.95648**	0.96306**	0.96079**	0.92568**	0.20571**	0	
YSHX	0.96020**	0.96784**	0.96376**	0.92623**	0.13063**	0.07236*	0

* p-values ≤ 0.05, ** p-values ≤ 0.01.

### Demographic inferences and divergence time

**Figure 5:**
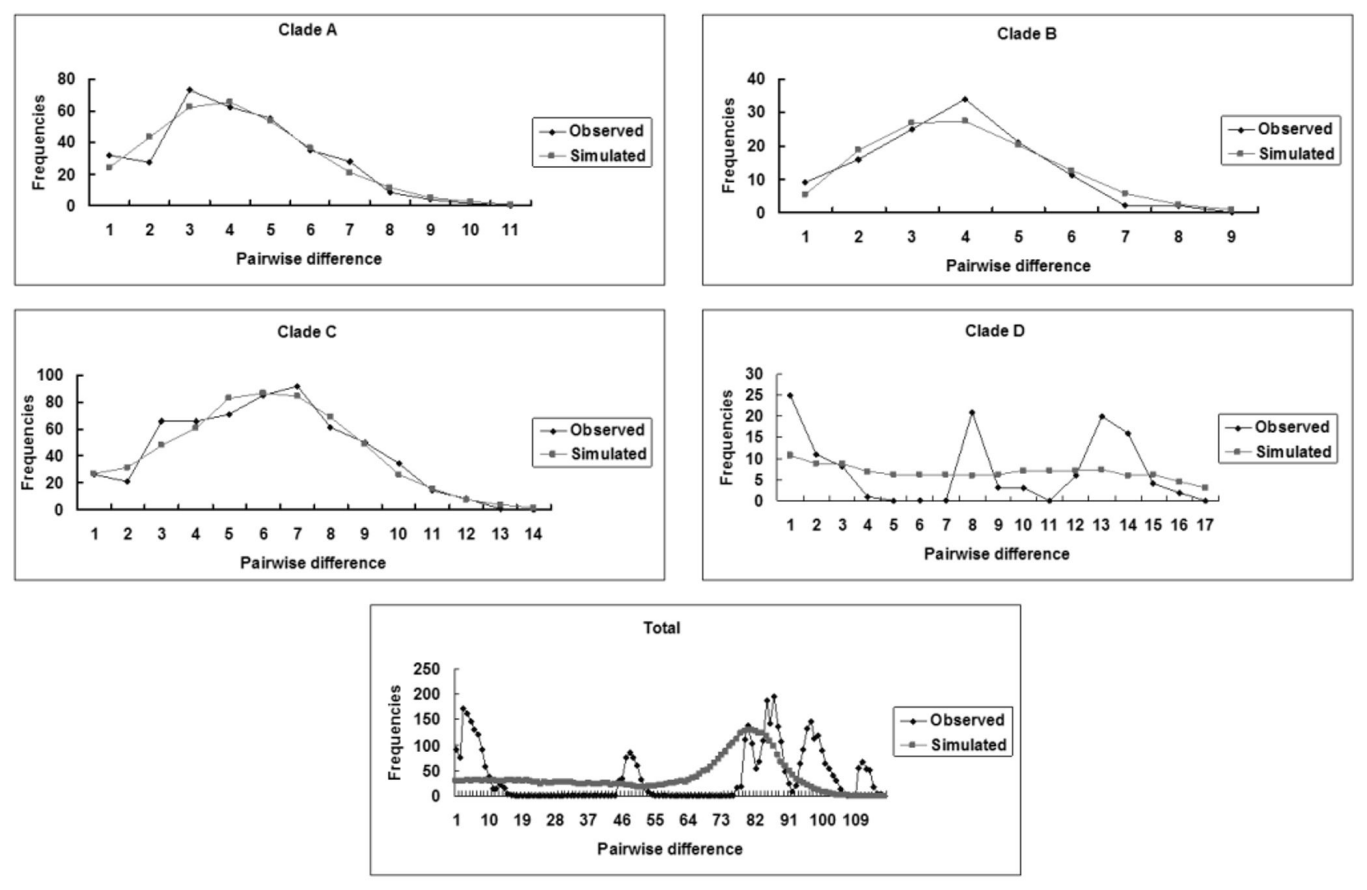
Mismatch distribution analysis for the total population and the clades. Clade A, the JGT–TCH population; Clade B, the HBSH population; Clade C, the YSHX–YXX–HSHX population; Clade D, the TTZH population. The line charts represent the observed frequences of pairwise differences among haplotypes.

**Figure 6:**
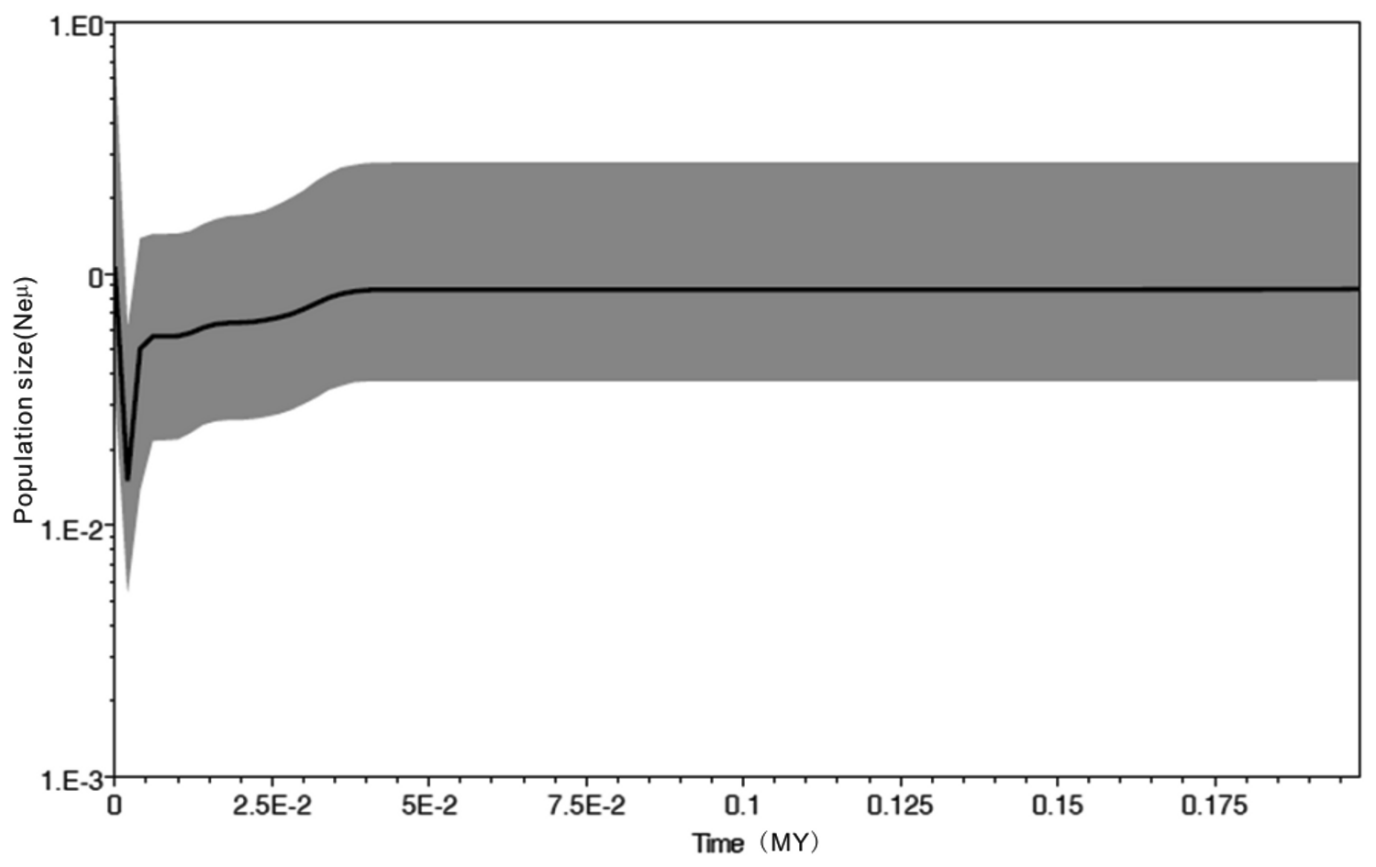
A Bayesian skyline plot derived from an alignment of mtDNA sequences of *Pachyhynobius shangchengensis* in China. The X-axis is in units of million years in the past and the Y-axis is Ne×μ (effective population size × mutation rate per site per generation). The median estimates are shown as thick solid lines, and the 95% HPD limits are shown by the gray areas.

The results of neutral test analyses of clade A, B, C indicated that both Tajima’s D and Fu’s *Fs* were negative, and some values have highly significant, except clade D possessed a positive value (Table 5). Mismatch distribution analyses showed a unimodal frequency distribution of pairwise differences in clade A, clade B and clade C (Figure 5). All above results suggest demographic expansion. The estimated expansion time of above clades was 0.06–0.03 Myr in the Late Pleistocene, the results were consistent with the analysis of the BSP (Table 5; Figure 6). And a sudden expansion was identified between 0.05–0.0025 Myr by BSP (Figure 6). However, both mismatch distribution analyses and the neutrality tests rejected a sudden population expansion in the clade D and total population (Table 5; Figure 5).

**Table 5:** Mismatch distribution analyses and neutrality tests.

Phylogroups	*τ*	T (MY)	Fu’s *Fs*	*p*-value	Tajima’s D	*p*-value
Clade A	3.52148	0.04025	-4.70975	< 0.01	-1.04370	0.14300
Clade B	2.98438	0.03411	-4.12564	< 0.01	-1.12491	0.12700
Clade C	5.38086	0.06150	-9.24512	0.00100	-1.45789	0.06500
Clade D	13.12109		1.99505	0.81300	0.58242	0.77600
Total population	90.18164		2.82270	0.82400	2.22060	0.98200

The results of analyses in the program BEAST inferred that the estimated age of the origin of *P. shangchengensis* on Mount Dabieshan in China to be 23.39 Myr. The divergence time between clade A and clade B was calculated to have taken place in the early Pleistocene 2.11 Myr, with a 95% highest posterior density (HPD) of 1.54–2.74 Myr. The divergence time between clade C and clade A+B was calculated to have taken place in the mid-Pliocene 3.93 Myr (95% HPD: 3.06–4.83 Myr). The divergence time between clade D and clade A+B+C was calculated to have taken place in the early Pliocene 4.93 Myr (95% HPD: 4.06–5.88 Myr) (Figure 3). 

## Discussion

### Pre-Pleistocene split and geologic history

The various drivers of species divergence associated with topography seem to play roles in the evolution of *P. shangchengensis*. In our study, we can see that phylogenetic analyses support four major clades. The median-joining network yielded four unconnected subnetworks corresponding to the four clades in the phylogenetic tree, and there are no shared haplotypes between clades (Figures 2, 3, 4 ). Such distributions of mitochondrial DNA haplotypes of *P. shangchengensis* may be interpreted as being the result of population isolation because of their specific biological habits. 

There are several reasons for Amphibians which are particularly sensitive to effects of topographic and altitudinal variation. Amphibians are generally highly site philopatric and poor dispersers [53–56]. Because of dessication and predation risks associated with terrestrial dispersal [57–59] and slow terrestrial locomotion [59], low vagility in amphibians is often attributed to dependence on moist habitats or wetland corridors for dispersal [59,60]. Thus, because of complex topography in the Mount Dabieshan regions, consequent range expansion and dispersal of individuals away from their natal sites are generally expected to be limited in *P. shangchengensis*. And this is the reason that *P. shangchengensis* can be found in patchy habitat on the Dabieshan Mountains [17,18,61].

Topography can drive divergence patterns [62], Pleistocene climatic fluctuations associated with cyclical glaciation events can drive divergence patterns too, even in lower latitudes [63,64]. China and its neighboring areas in East Asia have experienced a development of cooler and drier climates within the last 15 Myr, although most of China has never been covered by ice sheets [65]. Furthermore, tremendous climatic changes, particularly the Quaternary glaciations, have made many plants and animals extinct and influenced the evolution and distribution of many plants and animals in China and its neighboring areas, particularly during the Quaternary [66]. In our study, BEAST analysis showed that the estimated age of *P. shangchengensis* was 23.39 Myr (in the early Miocene), between the main clades, the divergence time taken place in the Pliocene, and by Bayesian skyline plot (BSP) a sudden expansion occurred in the Late Pleistocene. Therefore, climatic fluctuations probably impacted the distribution, demography and diversification of the species. We infer that *P. shangchengensis* which lived in the low lands disappeared during the interglacial in the Quaternary, because it could not adapt to hot and dry climate. By contrast, *Pachyhynobius shangchengensis*, which lived in higher elevations, survived because these areas were suitable for survival. Thus, the populations of *P. shangchengensis* have been isolated in the fragmented mountain habitats in the last interglacial to the present. This pattern also existed in other montane organisms [67,68]. 

### Conservation and management implications

The two goals of any conservation program are to maintain the genetic diversity of species for long-term evolutionary success and ensure their survival [20,69]. The number of *P. shangchengensis* decreases each year because of human activities such as arbitrary arrest or killing [17,19,61]. Therefore, *Pachyhynobius shangchengensis* should be protected.    

Management units (MUs) are commonly used designations for threatened or endangered taxa [70,71]. The data of our study can be used to establish MUs because these units are defined by either reciprocal monophyly in mtDNA or substantial allele frequency divergence at nuclear loci [70]. Thus, the four populations of *P. shangchengensis* can be considered MUs because genotypes in the four populations are closely related but not shared. Any conservation policy should concentrate on protecting the distinct populations with similar MUs similar to the conservation efforts for the Tibetan gazelle in China [15]. We would do our best to recommend to protect *P. shangchengensis* and avoid its extinction, we should take necessary measures, such as forbidding to kill any individual species in distribution areas of *P. shangchengensis.*

## Conclusions

In conclusion, *Pachyhynobius shangchengensis* has significant phylogeographic structure, the topography of the Dabieshan Mountains significantly affects the population genetic structure of it, and climatic oscillations during glacial periods in the Quaternary affected the distribution of this species. 
